# Antagonism of rhizosphere *Trichoderma brevicompactum* DTN19 against the pathogenic fungi causing corm rot in saffron (*Crocus sativus* L.) *in vitro*

**DOI:** 10.3389/fmicb.2024.1454670

**Published:** 2024-09-04

**Authors:** Li Tian, Xinyu Zhu, Yingqiu Guo, Qianjun Zhou, Lili Wang, Wankui Li

**Affiliations:** Key Laboratory of New Resources and Quality Evaluation of Traditional Chinese Medicine State Administration of Traditional Chinese Medicine, Institute of Traditional Chinese Medicine, Shanghai University of Traditional Chinese Medicine, Shanghai, China

**Keywords:** *Trichoderma brevicompactum*, saffron, rhizosphere, PGPR, antagonistic activity, biological control

## Abstract

**Introduction:**

Corm rot in saffron (*Crocus sativus* L.) significantly impacts yield and quality. Non-toxic fungi, particularly *Trichoderma* species, are valuable for biological control due to their production of diverse and biologically active secondary metabolites.

**Methods:**

This study aimed to isolate an effective antagonistic fungus against the pathogenic fungi causing corm rot in saffron. Four pathogenic fungi (*Fusarium oxysporum*, *Fusarium solani*, *Penicillium citreosulfuratum*, and *Penicillium citrinum*) were isolated from diseased saffron bulbs in Chongming. Initial screening through dual culture with these pathogens re-screening from rhizosphere soil samples of *C. sativus* based on its inhibitory effects through volatile, nonvolatile, and fermentation broth metabolites. The inhibitory effect of biocontrol fungi on pathogenic fungi in vitro was evaluated by morphological observation and molecular biology methods.

**Results:**

Antagonistic fungi were identified as *Trichoderma brevicompactum* DTN19. *F. oxysporum* was identified as the most severe pathogen. SEM (scanning electron microscope) and TEM (transmission electron microscope) observations revealed that *T. brevicompactum* DTN19 significantly inhibited the growth and development of *F. oxysporum* mycelium, disrupting its physiological structure and spore formation. Additionally, *T. brevicompactum* DTN19 demonstrated nitrogen fixation and production of cellulase, IAA (Indole acetic acid), and siderophores. Whole-genome sequencing of strain DTN19 revealed genes encoding protease, cellulase, chitinase, β-glucosidase, siderophore, nitrogen cycle, and sulfate transporter-related proteins

**Discussion:**

*T. brevicompactum* DTN19 may inhibit the propagation of pathogenic fungi by destroying their cell walls or producing antibiotics. It can also produce IAA and iron carriers, which have the potential to promote plant growth. Overall, *T. brevicompactum* DTN19 showed the development prospect of biological agents.

## 1 Introduction

Saffron is the dried stigma of *C. sativus* L., which belongs to the Iridaceae family. It is native to Iran, Greece, India, and Spain and is a world-famous dye and spice. In China, Saffron is a Chinese herbal medicine with the homology of medicine and food, which was later included in the system of traditional Chinese medicine resources, and is known as “red gold.” It is sweet in taste, flat in nature, heart-warming, and liver meridian, saffron also has blood activation, stasis dissolution, blood cooling, detoxifying, nerve tranquilizing, and antidepressant effects (Mzabri et al., 2019; Cardone et al., 2020; Zhao et al., 2021). People’s demand for saffron is increasing day by day because of its therapeutic properties. However, saffron is a triploid plant and can only rely on bulbs for vegetative reproduction. Therefore, bulbs are the material basis for the flowering and formation of progeny bulbs. In the natural environment, bulbs, as underground organs, are constantly besieged by a variety of potentially pathogenic organisms, which has caused the crocus planting area to shrink year by year in many countries, becoming one of the limiting factors for the sustainable development of the global saffron resource industry (Rasool et al., 2021; Abu-Izneid et al., 2022; Mirghasempour et al., 2022).

Since the successful cultivation of crocus in Chongming, China, in the 1980s, large-scale planting has expanded to Shanghai, Zhejiang, Jiangsu, Anhui, and Henan. Two cultivation modes have emerged: a “two-stage” indoor flowering mode in Shanghai and Zhejiang, and a “one-stage” open-field flowering mode in Henan and Anhui. In 2021, large-scale flowering abnormalities broke out in major crocus planting areas in my country such as Chongming in Shanghai, Jiande in Zhejiang, and Bozhou in Anhui, resulting in a significant reduction in saffron production, with the yield per mu only accounting for about one-third of that in normal years. Among them, the crocus planting area and crocus production in Chongming decreased from 1,062 mu and about 438 kg to 357 mu and about 145 kg, respectively. In the early stage of this study, we confirmed that four strains of *F. oxysporum, F. solani, P. citreosulfuratum,* and *P. citrinum* were the main pathogens of saffron corm rot in Chongming Island—one of the main saffron-producing areas in China—among which *P. citreosulfuratum* was first reported to cause the black spot disease of saffron (Hu et al., 2022). The problem of corm rot has seriously affected the sustainable development of my country’s saffron resource industry. Therefore, it is necessary to actively respond and find practical prevention and control methods.

Microorganisms growing in the rhizosphere of medicinal plants have long been affected by substances secreted from the roots of medicinal plants, and their microbial species and the proportion of bioactive substances produced are relatively high (Yuan et al., 2022). A large number of research results show that *Trichoderma* has antagonistic activity against a variety of plant pathogens, and has a strong colonization ability, which can play a biological control role through various mechanisms such as competition, heavy parasitism, antibiotic, and synergistic antagonism (Bononi et al., 2020; Khan and Javaid, 2020). For example, *Trichoderma gamsii* showed mycoparasitic activities as well as antibiosis against phytopathogens (Chen et al., 2016). *Trichoderma koningiopsis* PSU 3-2 can compete for nutrients and space to produce VOCs (antibiosis) and CWDEs (mycoparasitism) to control pepper anthracnose (Ruangwong et al., 2021). Recent works have shown that common plant diseases such as root rot disease, damping off, fruit rot, and other plant diseases can be controlled by *Trichoderma* spp. (Elshahawy and El-Mohamedy, 2019; Javaid et al., 2021). This study collected healthy crocus rhizosphere soil samples from Chongming, screened for fungi with antagonistic activity against corm rot, and preliminarily evaluated their antibacterial ability, providing potential biological control options for saffron corm rot pathogens.

## 2 Materials and methods

### 2.1 Indicative pathogenic fungi

In 2020, four pathogenic fungi (*F. oxysporum, F. solani, P. citreosulfuratum,* and *P. citrinum*) were isolated and purified from the rotten bulbs of saffron, and were stored in our laboratory’s 4°C refrigerator.

### 2.2 Collection of soil and isolation and purification of biocontrol fungus

Rhizosphere soil samples were collected from the *C. sativus* planting base in Chongming District, Shanghai, in March 2023. The soil was air-dried in the shade, ground with a mortar, and sieved through a 20-mesh sieve. Ten grams of soil were weighed and placed in a flask containing 90 mL of sterile water, which was shaken at 28°C and 150 rpm for 2 h. Subsequently, 1 mL of the mixture was transferred to a centrifuge tube with 9 mL of sterile water and shaken to create a 10^−2^ soil dilution. This process was repeated to prepare 10^−3^, 10^−4^, and 10^−5^ soil suspensions. Next, 200 μL of each suspension was added to modified Martin medium, Bengal red medium, PDA medium, and MEA medium, each containing penicillin potassium (150 mg/L) and streptomycin sulfate (120 mg/L) to cultivate fungi (Thi Minh Le et al., 2019). The procedure was repeated three times. After evenly coating the Petri dishes, they were incubated at 28°C for 7 days. Fungal colony growth was observed daily, and colonies were promptly transferred to PDA medium. After purification, the fungi were inoculated into slant culture media and stored at 4°C or frozen with glycerol at −80°C for future use.

### 2.3 Preliminary screening of biocontrol fungi

Preliminary screening of isolated fungi using plate confrontation method (Lu et al., 2020). A hole punch was used to make pathogen plugs with a diameter of 5 mm at the edge of *F. oxysporum*, and *F. solani* colonies, and the preliminarily obtained fungi cultured for 7 days. The preliminarily *P. citreosulfuratum* and *P. citrinum* were inoculated on a PDA plate for 2 days. The pathogen plugs were placed in the center of the PDA plate, and then the separated fungi were inoculated approximately 2.5 cm away from the fungal cake in a crisscross formation and compared with the PDA plate with only pathogen plugs. Subsequently, the plates were incubated at 28°C for 5–10 days, and the appearance of antagonistic bands on the experimental plates was observed. Each fungus was tested in triplicate, and the entire experiment was repeated three times. The inhibition rate was calculated to identify the most effective antagonistic fungus. Inhibition rate = (Diameter of the colony in the control group - Diameter of the colony in the treatment group / Diameter of the colony in the control group × 100%) (Saravanakumar and Wang, 2020).

### 2.4 Re-screening of biocontrol fungi

#### 2.4.1 Copyright inhibitory effects of *Trichoderma* volatile metabolites on pathogenic fungus

The disk filter membrane method was used to determine the antibacterial activity of the non-volatile metabolites of *Trichoderma* (Wang et al., 2023). The cellophane was cut into disks with a diameter of 9 cm, sterilized by high-pressure steam, and stuck in the center of the PDA culture medium (*d* = 9 cm). *Trichoderma* plugs were inoculated in the center of the cellophane-covered culture media and incubated at 28°C in the dark. When *Trichoderma* reached the edge of the cellophane, the cellophane and the entire colony were removed, and plugs of the four pathogenic fungi were inoculated in the center of the Petri dishes. The pathogen was inoculated into an untreated PDA medium as a control, and each treatment and control were set up in triplicate. Observe the growth status of each pathogen every day, measure the diameter of the pathogen take photos on the third day, and calculate the bacteriostatic effect according to the growth inhibition rate formula.

#### 2.4.2 Inhibitory effects of *Trichoderma* refractory metabolites on pathogenic

The plate buckle method was used to determine the antibacterial activity of *Trichoderma* volatile metabolites (Sornakili et al., 2020). A 5 mm *Trichoderma* plug was inoculated into PDA culture medium and incubated at 28°C for 3 days. Subsequently, a 5 mm plug of the pathogenic fungi was inoculated onto a separate PDA culture medium. The plate with the pathogenic fungi was then placed inverted on top of the *Trichoderma* plate, with a layer of cellophane between them. This setup was incubated at 28°C for 7 days. Calculate the inhibition rate compared with the PDA plate inoculated with pathogenic fungi only.

#### 2.4.3 HPLC detection of secondary metabolites in *Trichoderma* fermentation broth and determination of their antibacterial activity

A sterilized 5 mm punch was used to obtain *Trichoderma* plugs from an activated PDA plate. These plugs were then inoculated into a triangular flask containing 200 mL of medium, with a total of 10 flasks prepared. The flasks were placed on a shaker at 28°C and 150 rpm for 5 days. After incubation, the hyphae were filtered using sterile absorbent cotton and then sterile filter paper. An equal volume of ethyl acetate was added to the filtered fungal liquid, soaked for 24 h, and extracted three times per flask. The extracts were combined and concentrated at 45°C using a rotary evaporator to obtain the crude fermentation extract.

The crude extract was dissolved in methanol to prepare a 10 mg/mL solution, which was analyzed by HPLC using a C18 column (5 μm, 4.6 mm × 250 mm). The mobile phase consisted of an acetonitrile-water system with 0.1% formic acid added to the water. The analysis conditions were as follows: 0–5 min, 10 to 30% acetonitrile; 5–40 min, 30 to 90% acetonitrile; 40–55 min, 90 to 10% acetonitrile; 55–60 min, 10% acetonitrile. The flow rate was 1.0 mL/min, and detection was performed at 210 nm.

The crude extract was dissolved in DMSO to prepare a 10 mg/mL solution. Pathogenic fungi were inoculated in the center of a PDA culture dish, and holes were punched 2.5 cm away from the fungal cake in a crisscross pattern. The crude extract solution was added to these holes, while a DMSO solution of the same volume served as the control. The Petri dishes were incubated at 28°C in the dark, with each treatment replicated three times. The mycelial growth of the pathogenic fungi was recorded daily, and the diameter of the colonies was measured and photographed on the third day to calculate the inhibition effect.

### 2.5 Morphological characteristics of antagonistic fungi DTN19

Antagonistic fungi were inoculated on PDA culture medium and incubated at 28°C for 5–10 days to observe the morphological characteristics of the colonies. A small number of hyphae were picked with an inoculation needle and placed on a glass slide with a drop of distilled water. The sample was mixed evenly, gently covered with a coverslip, and excess water was absorbed with absorbent paper. The morphological characteristics of hyphae and spores were then observed under an optical microscope (OM).

A square agar block (approximately 0.5 cm side length) containing *Trichoderma* was cut from the culture medium, fixed in 2.5% glutaraldehyde at 4°C for 24 h, and rinsed three times with 0.1 M phosphate buffer (pH 7.0) for 15 min each. The sample was then fixed with 1% osmium tetroxide for 1–2 h. After carefully removing the osmium tetroxide, the sample was rinsed three times with 0.1 M phosphate buffer (pH 7.0) for 15 min each. The samples were dehydrated using a graded ethanol series (30, 50, 70, 80, 90, and 95%), each for 15 min, followed by two treatments with 100% ethanol for 20 min each. The sample was then treated with a 1:1 mixture of ethanol and isoamyl acetate for 30 min, followed by pure isoamyl acetate for 1 h or overnight.

### 2.6 Molecular identification of biocontrol fungi

The general primers ITS1 (5′-TCCGTAGGTGACCTGCGG-3′) and ITS4 (5′-TCCTCCTCCGGTTATGATTGC-3′) were selected to amplify the DNA of antagonistic fungi. The amplified PCR products were then purified and Sanger sequenced in Shanghai Meiji Biotechnology Co., Ltd. The ITS sequence was compared to the NCBI GenBank^1^ database using the blast^2^ (Bahadou et al., 2018), algorithm. A phylogenetic tree was then constructed using the adjacency method in the MEGA 7.0 software (Agarwal et al., 2017).

### 2.7 Effects of biocontrol fungi on mycelial morphology of *Fusarium oxysporum*

Agar blocks (5 mm × 5 mm) from the junction of two colonies growing on the PDA plate were taken to observe their microscopic characteristics under a scanning electron microscope (SEM) using the aforementioned method. A 5 mm^3^ sample of mycelium was prepared for transmission electron microscopy (TEM) following the method described by Aboelnour et al. (2020). Finally, cut them into 70 nm slices with Leica 705,902 ultrathin slicer (Germany), dye them with lead citrate for 15 min, rinse them with dd H_2_O three times, and naturally dry them before examining them with FEI Tecnai G2 alcohol TEM.

### 2.8 Hydrolase activity determination and PGP potential assessment

Among all known biological control mechanisms, the secretion of lyase is considered to be an effective method to prevent plant pathogens near the rhizosphere. The activity of chitinase and glucanase produced by the strain were detected by connecting the selected activated biocontrol fungi to the chitinase assay plate and glucanase assay plate, respectively (Aoki et al., 2020; Win et al., 2021). According to the protease assay plate prepared by Rodarte et al. (2011), the glucanase activity of the strain was determined. A cellulase assay plate was prepared according to the method of Sallam et al. (2021), then an appropriate amount of 1 mg/mL Congo red solution was added to the Petri dish, dyed for 1 h, the dye solution was poured out, and an appropriate amount of 1 mol/L NaCl solution was added for decolorization for 1 h; to determine the activity of cellulase produced by the strain.

The phosphate solubilizing ability was qualitatively determined by inoculating biocontrol fungi in the American Botanical Research Institute’s phosphate growth medium (NBRIP) and culturing for 5 days (Saravanan et al., 2004). Biocontrol fungi were inoculated on Ashby nitrogen-free medium plates and cultured for 5 days, and the nitrogen-fixing ability was determined by observing the colonies (Cui et al., 2022). The biocontrol fungi were inoculated on silicate culture medium plate for 5 days, and their potassium-dissolving ability was examined (Lue and Huang, 2010). Using the method of Salkowski colorimetry (Kumar et al., 2017), the biocontrol fungi were inoculated into King’s B culture medium containing inducer tryptophan and without tryptophan respectively, and liquid fermentation was carried out at 28°C for 7 days, with three repetitions, and 2 mL of fermentation medium was centrifuged at 12,000 r/min for 15 min, and 1 mL of supernatant was placed in a centrifuge tube. Add the same amount of Salkowski colorimetric solution (50 mL of 35% HClO_4_ + 1 mL of 0.5 mol/L FeCl_3_), place the test tube in the dark for 30 min, and then observe the IAA-producing ability of the strain. Inoculating biocontrol fungi in nitrogen-free liquid culture medium for 3 days, inoculating 5% inoculum in DF culture medium for 3 days, and then inoculating 5% inoculum in ADF culture medium for 3 days; Normal growth indicates the production of ACC (1- aminocyclopropane -1- carboxylic acid) deaminase (Etesami and Alikhani, 2017). The ability of the iron-producing carrier was determined by the MAS-CAS method. The mycelium of the strain to be tested was inoculated on CAS agar medium and cultured for 7 days at 28°C in the dark. By observing the color changes around fungal colonies on CAS agar, the colonies are surrounded by orange-yellow bands on the plate and are considered to be iron-producing carriers (Konsue et al., 2020). The ability of fungi to produce ammonia in peptone water was determined (Khalil et al., 2021).

### 2.9 Whole genome sequencing of biocontrol fungi DTN19

#### 2.9.1 Sample preparation

Strain DTN19 was inoculated into PDA liquid culture medium, cultured at 200 r/min and 28°C for 48 h, then the mycelium was quickly frozen in liquid nitrogen for 15 min, put into a self-sealed bag, and immediately transported to Meiji Biomedical Technology Co., Ltd. for genome sequencing.

#### 2.9.2 DNA extraction, genome sequencing, and assembly

Genomic DNA was extracted using the Wizard Genomic DNA Purification Kit (Promega, United States). The purified genomic DNA was quantified, and the genome was sequenced by PacBio Sequel IIe and Illumina sequencing. The data obtained by sequencing were analyzed by subsequent bioinformatics. Cloud.majorbio.com of Shanghai Major Bio-pharm Technology Co., Ltd. was used for all analyses.

#### 2.9.3 Gene prediction and functional annotation

Prediction of open-read frames (ORFs) was performed using Maker2, tRNA-scan-SE was used for tRNA prediction and Barrnap was used for rRNA prediction. The predicted ORFs were annotated through NR, Swiss-Prot, Pfam, GO, COG, KEGG, and CAZY databases using sequence alignment tools such as BLAST, Diamond, and HMMER. Briefly, each set of query proteins was aligned with the databases, and annotations of best-matched subjects (*E*-value < 10^−5^) were obtained for gene annotation.

## 3 Results

### 3.1 Preliminary screening of biocontrol fungi

**Figure 1:**
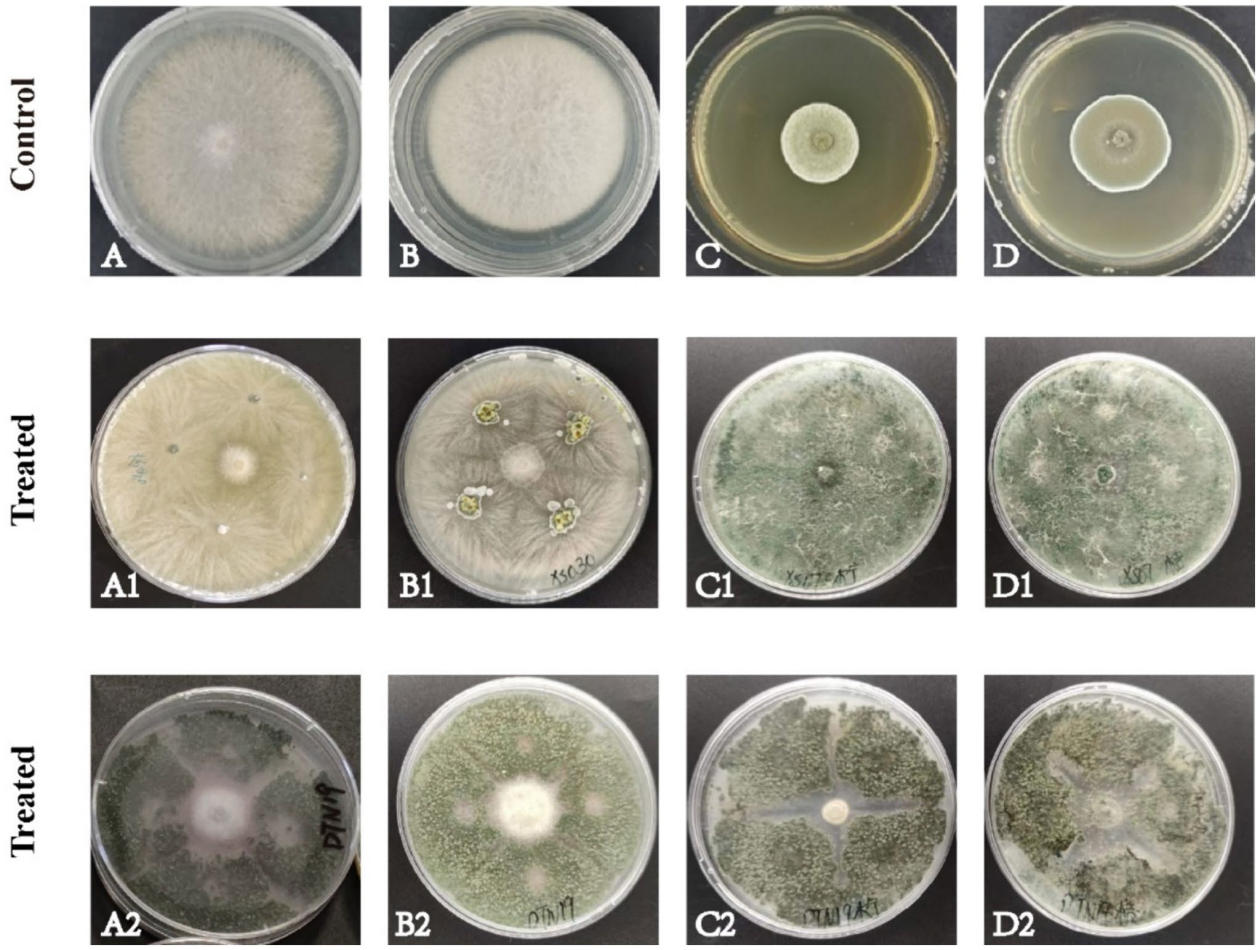
Antagonism of the strain on four pathogenic fungi in PDA culture medium for about 7 d. **(A)** *F. oxysporum*; **(B)** *F. solani*; **(C)** *P. citreosulfuratum*; **(D)** *P. citrinum*; (1) Strain XS030; (2) Strain DTN19.

In this experiment, 138 strains of fungi were isolated from the rhizosphere soil of *C. sativus*. Two strains of fungi DTN19 and XS030 with good antagonistic effects were screened, and their inhibitory rates against *F. oxysporum, F. solani, P. citreosulfuratum,* and *P. citrinum* were 77.56, 76.71, 71.93 and 65.10%, respectively. 79.49%, 79.45%, 78.07%, 76.67% (Figure 1).

### 3.2 Rescreening of biocontrol fungi

**Figure 2:**
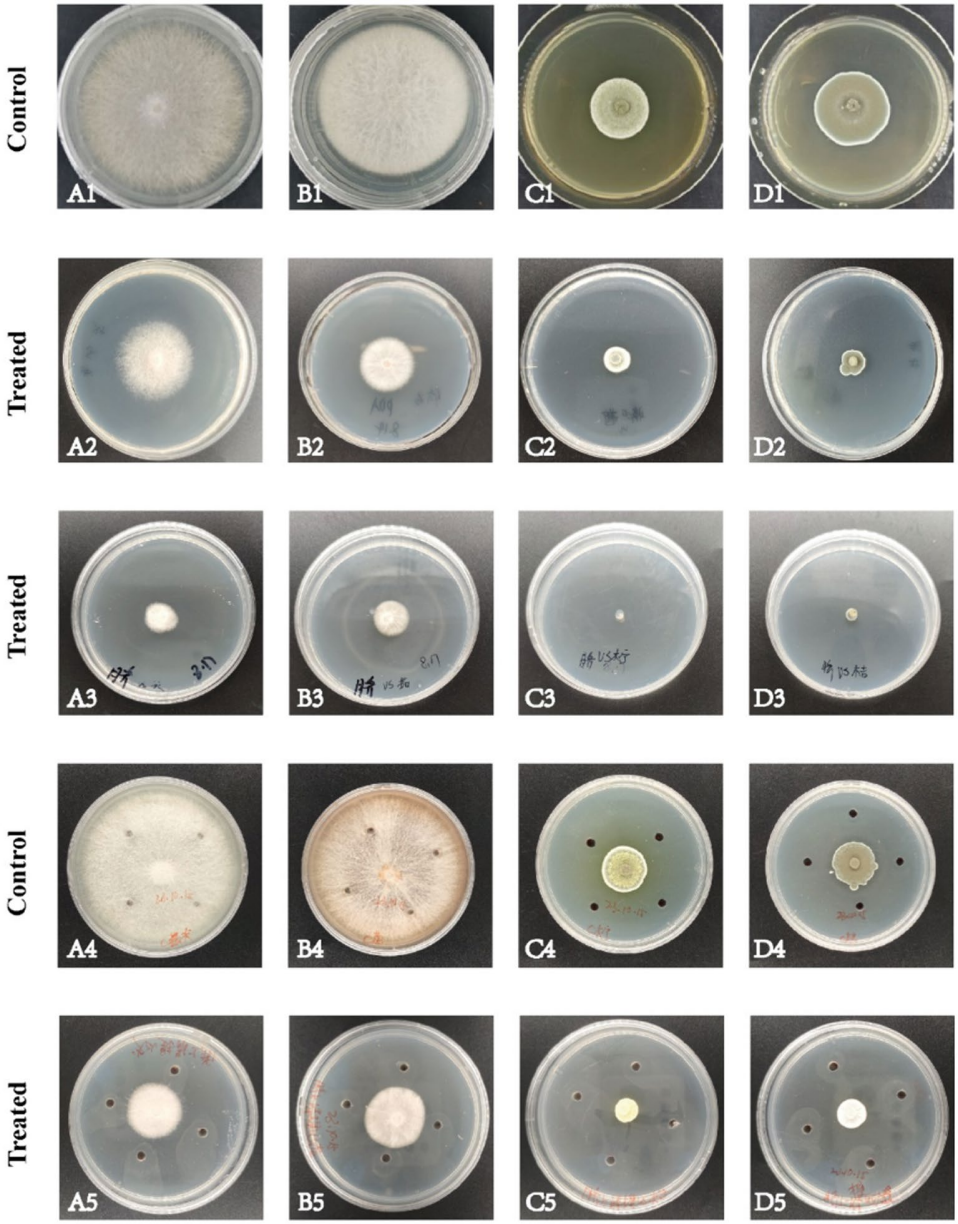
Antifungal activities of volatile metabolites, nonvolatile metabolites and fermentation broth extract. **(A)** *F. oxysporum*; **(B)** *F. solani*; **(C)** *P. citreosulfuratum*; **(D)** *P. citrinum*; (1) Control; (2) Volatile metabolites; (3) Non-volatile metabolites; (4) Control; (5) Fermentation broth extract.

**Figure 3:**
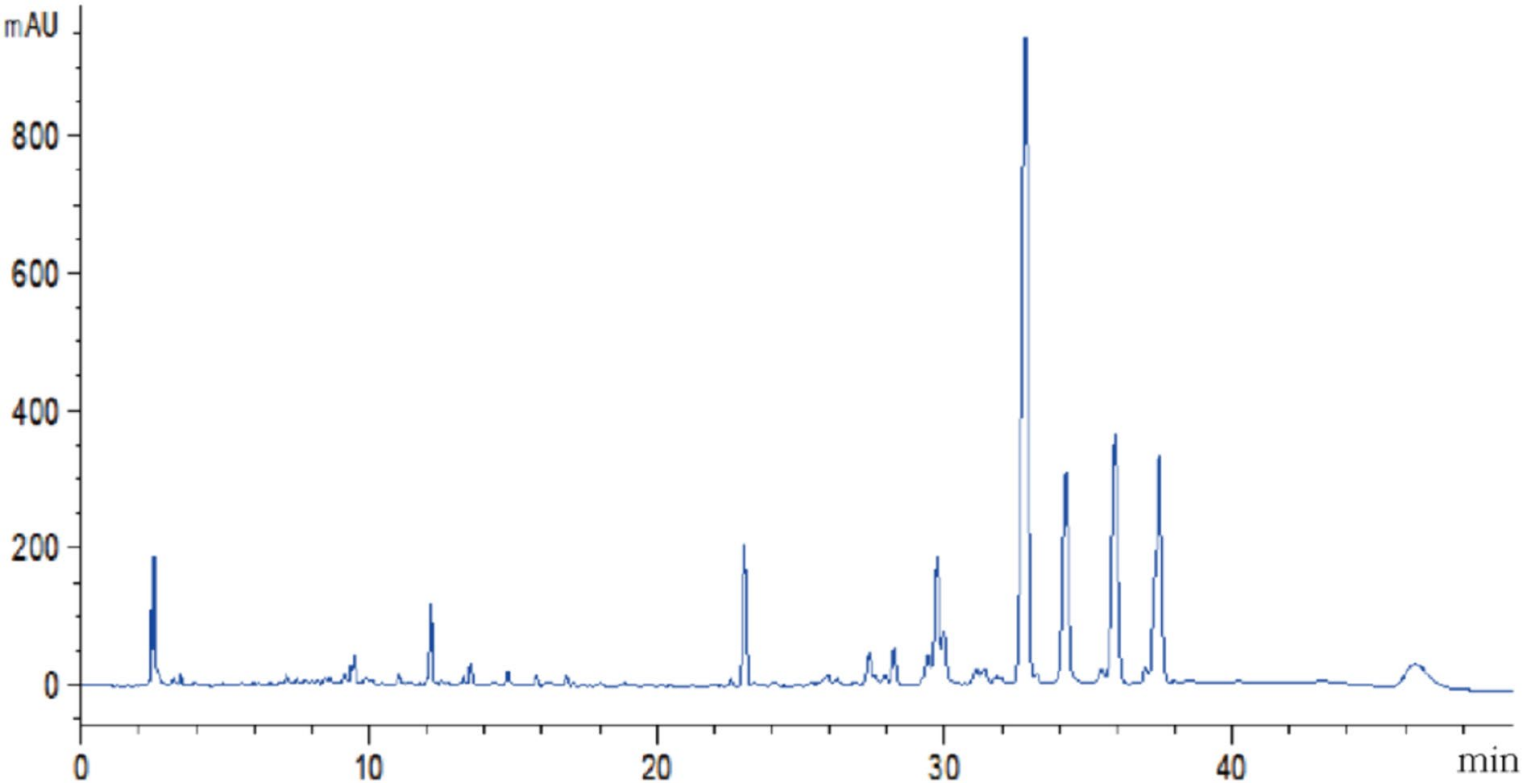
HPLC analysis of chemical diversity in crude organic extracts.

The volatile and nonvolatile metabolites of strain DTN19, as well as the crude extract obtained via rotary evaporation after ethyl acetate extraction, exhibit significant inhibitory effects. Inhibitory rates against *F. oxysporum, F. solani*, *P. citreosulfuratum,* and *P. citrinum* are detailed in Supplementary Table S1 and Figure 2. The chemical diversity of crude extract from the fermentation broth of strain DTN19 detected by HPLC is shown in Figure 3. Both the comprehensive activity test and HPLC analysis demonstrate that the strain possesses substantial biological activity and a rich array of metabolites, highlighting its strong research potential.

### 3.3 Identification of biocontrol fungus DTN19

**Figure 4:**
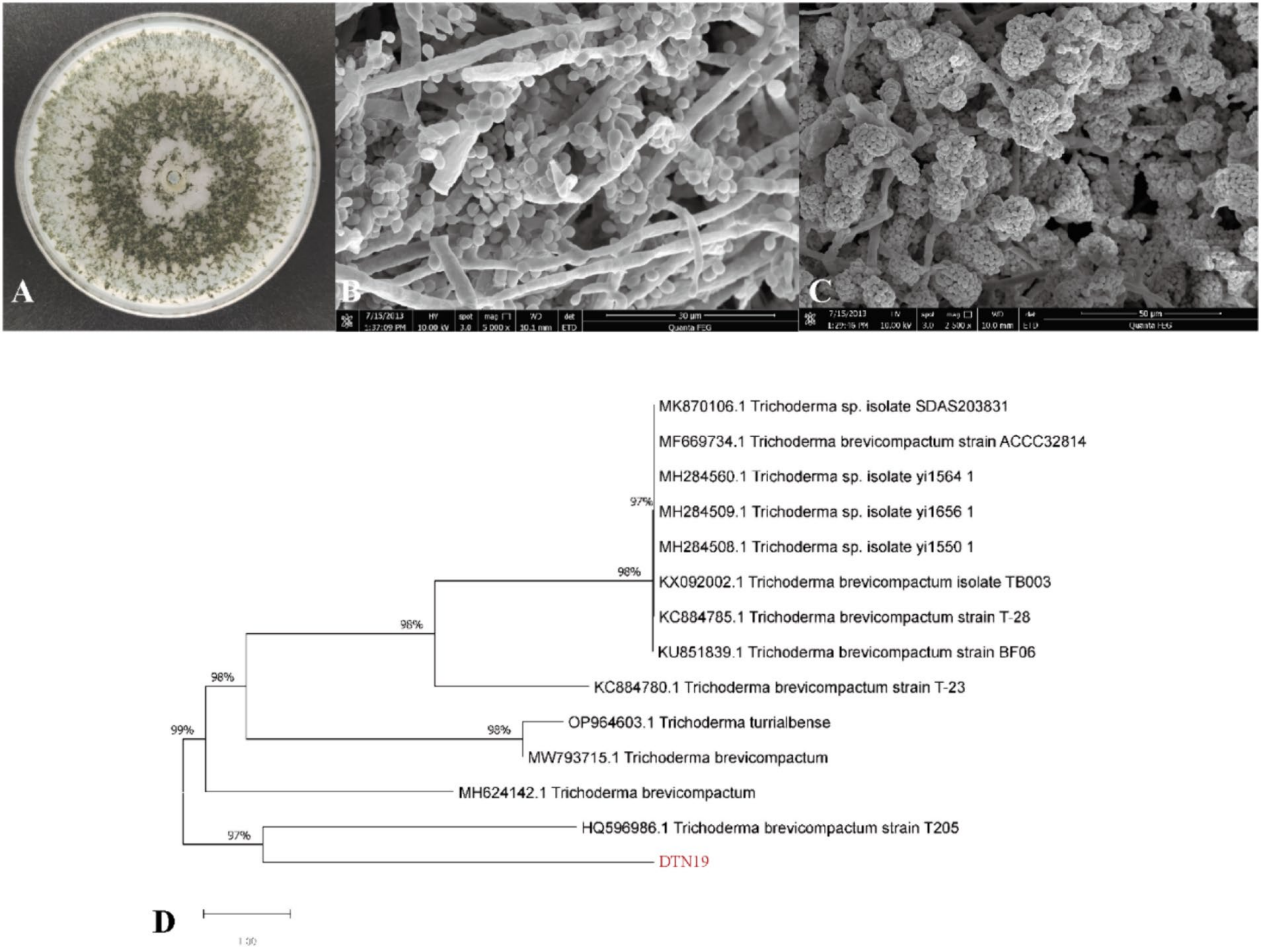
Strain DTN19 identification. **(A)** Colonial morphology; **(B,C)** Microscopic morphology; **(D)** Phylogenetic tree of 18SrDNA sequence of fungi.

The aerial mycelium of strain DTN19 grows extensively on PDA plates, forming fluffy, felt-like colonies that are white, thick, and prominent with obvious concentric circles. After 5 days, the PDA plate is covered with numerous dark green spores, while the reverse side shows green and brown pigmentation. The conidiophores are thick, with short main axes and primary branches, and few terminal branches. The swollen, relatively short conidiophores have 2–4 rounds. Sporogenous cells are ampoule-shaped with an enlarged base and constricted neck, forming compact clusters of conidia. Conidia are oval and smooth, with a size of 2.4–2.8 μm × 2.0–2.4 μm (Figures 4A–C). According to the cultural and morphological characteristics of strain DTN19, strain DTN19 was initially identified as *T. brevicompactum*. Then, according to the ITS phylogenetic tree (Figure 4D), strain DTN19 was proved to be *T. brevicompactum* from the molecular biological level, with the accession number OR680934.

### 3.4 Effect of biocontrol fungi DTN19 on morphology of *Fusarium oxysporum*

**Figure 5:**
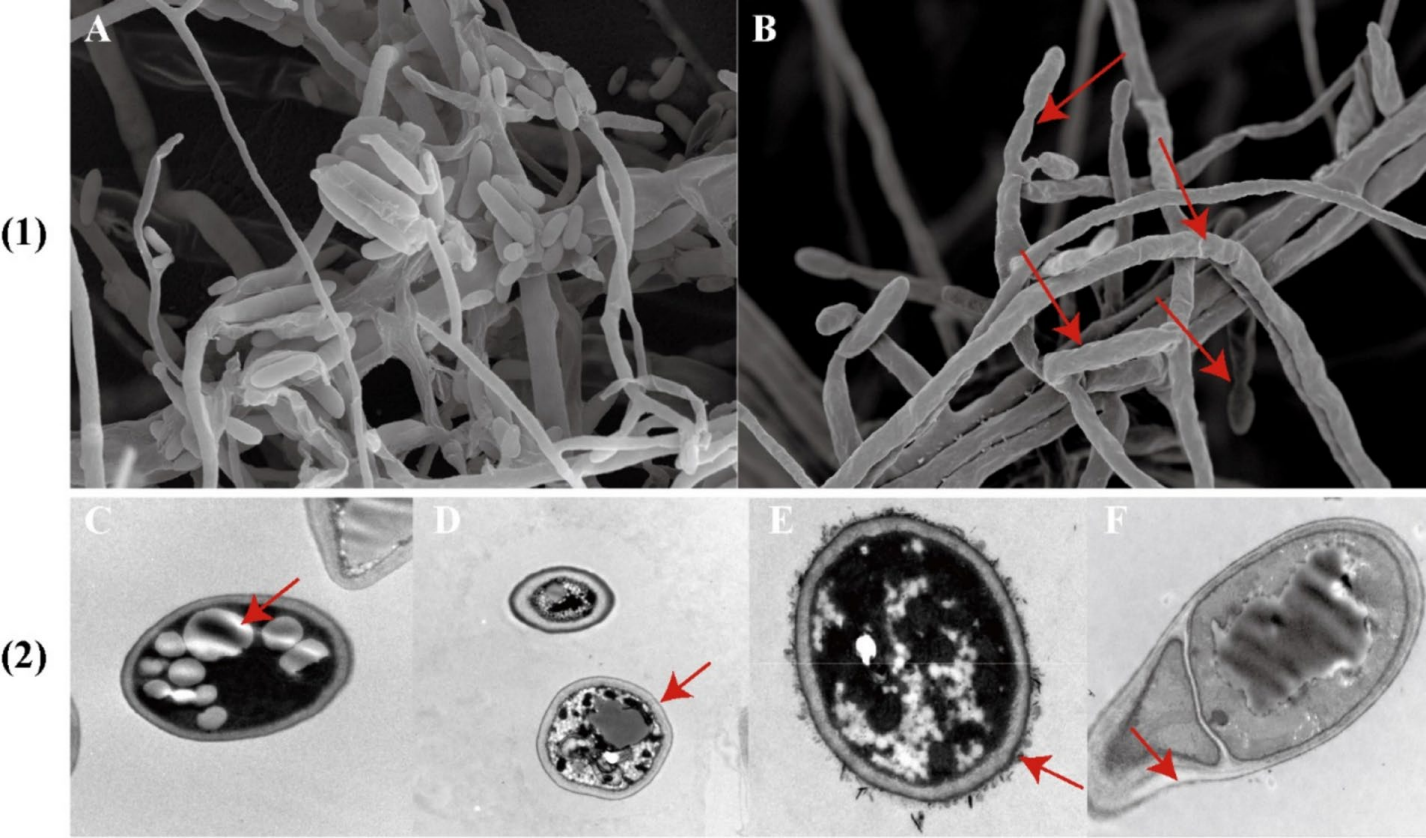
(1) Observe the antibacterial effect by SEM; (2) TEM antibacterial effect was observed; **(A)** Contrast; **(B)** Experimental; **(C,D)** Contrast; **(E,F)** Experimental.

As shown in Figure 5, the control group exhibits hyphae are full, with complete morphological structure, smooth surfaces, uniform thickness, and intact, compact cell walls and membranes. The cytoplasm is evenly distributed, and the conidia are spindle-shaped and display robust growth. In contrast, *F. oxysporum* affected by strain DTN19 shows broken mycelium with an uneven surface, abnormal development at the tips, and deformed, reduced conidia. The cell walls are ruptured, leading to leakage of cellular contents, decreased vacuoles, and a turbid cytoplasm.

### 3.5 Analysis of degradation enzyme production and growth-promoting properties of biocontrol fungus DTN19

According to the experimental phenomenon, strain DTN19 shows strong cellulase activity, can fix nitrogen, and produces IAA and siderophores, as shown in Supplementary Table S2.

### 3.6 Genome sequencing and assembly

#### 3.6.1 Gene function annotation

In this study, the whole genome of strain DTN19 was sequenced by the Illumina NovaSeq sequencing platform. The gene prediction results showed that there were 11,437 protein-coding genes in the genome of DTN19, with a total gene sequence length of 22,637,557 bp, an average length of coding genes of 1979.33 bp and a G + C content of 51.53%, as shown in Supplementary Table S3.

#### 3.6.2 GO function annotation

**Figure 6:**
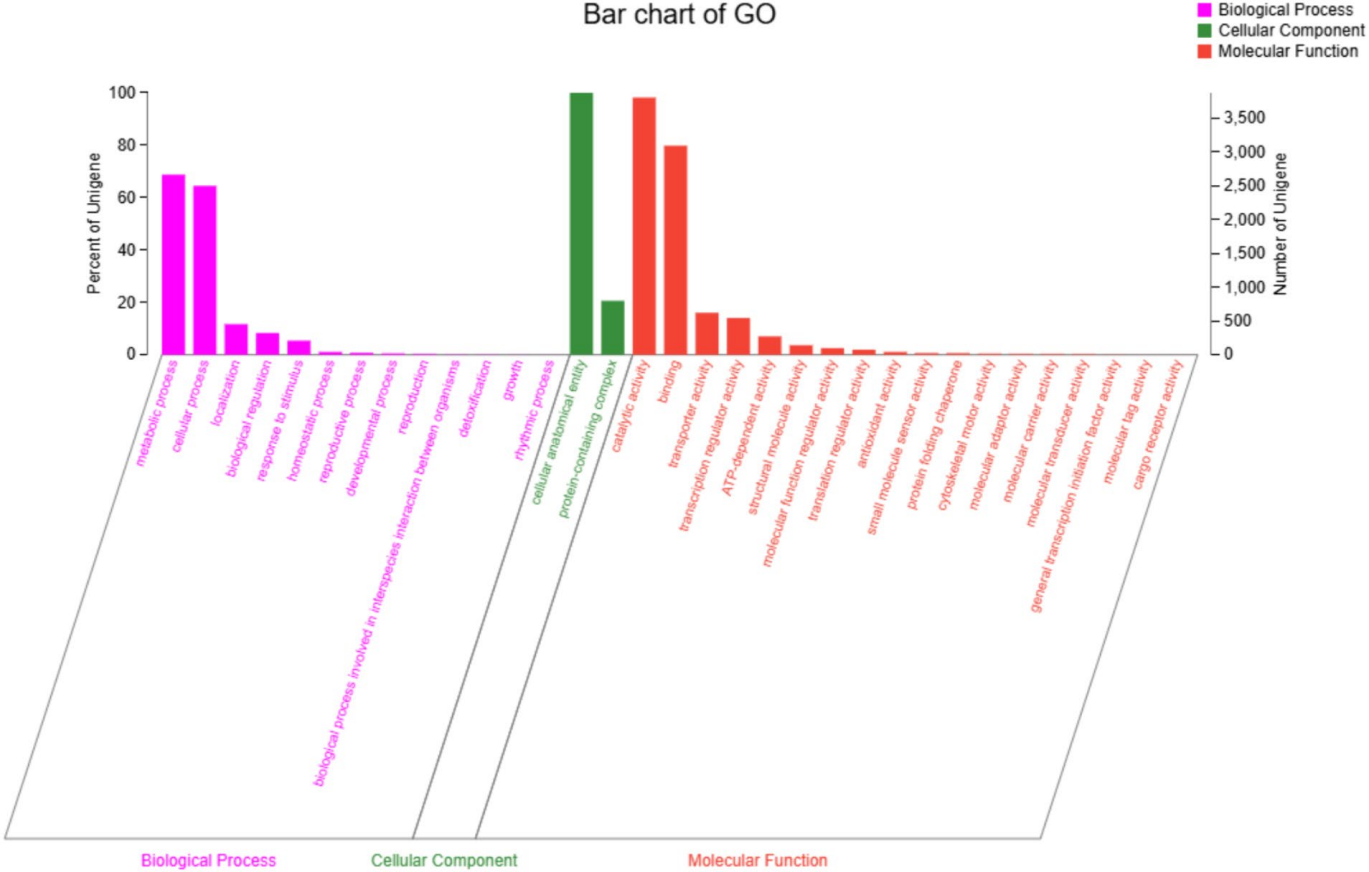
GO function classification of strain DTN19.

A total of 7,447 genes were obtained from the genome of strain DTN19 by lnterPro annotation, accounting for 65.11% of the total protein coding gene sequence (Figure 6). The gene function can be divided into three subcategories: 13 branches of biological process, 2 branches of molecular function, and 18 branches of cellular components. Metabolic process (GO: 0008152) and cellular process (GO: 0009987) are the most involved genes in biological process subcategories, with 2,666 and 2,501 genes, respectively. Cellular anatomical entity (GO: 0110165) is the most involved gene in the subcategory of cell components, reaching 3,878. The molecular functional subclasses are catalytic activity (GO: 0003824) and binding (GO: 000548), with 3,808 and 3,095 genes, respectively.

#### 3.6.3 KEGG function annotation

**Figure 7:**
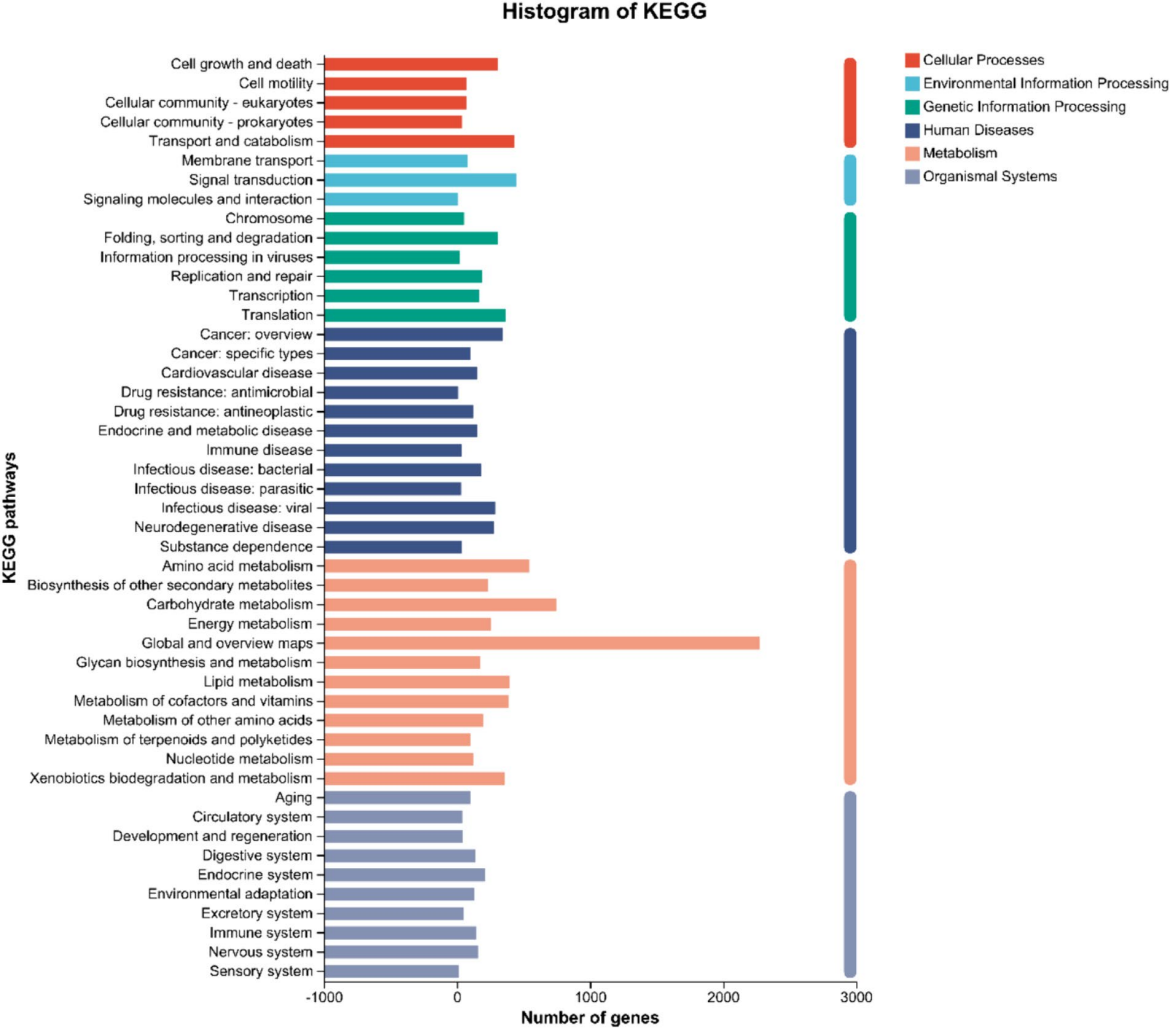
KEGG function classification of strain DTN19.

KEGG Pathway annotation of strain DTN19 identified 7,220 genes associated with metabolic pathways, which were distributed across 48 pathways. There are seven metabolic pathways involving a large number of genes: Global and overview maps, with a total of 2,276 genes; Carbohydrate metabolism, a total of 748 genes; Amino acid metabolism, a total of 544 genes; Signal transduction, a total of 445 genes; Transport and catabolism, a total of 429 genes; Lipid metabolism, a total of 397 genes; Metabolism of cofactors and vitamins, 390 genes in total, Translation, 367 genes in total (Figure 7).

#### 3.6.4 COG function annotation

**Figure 8:**
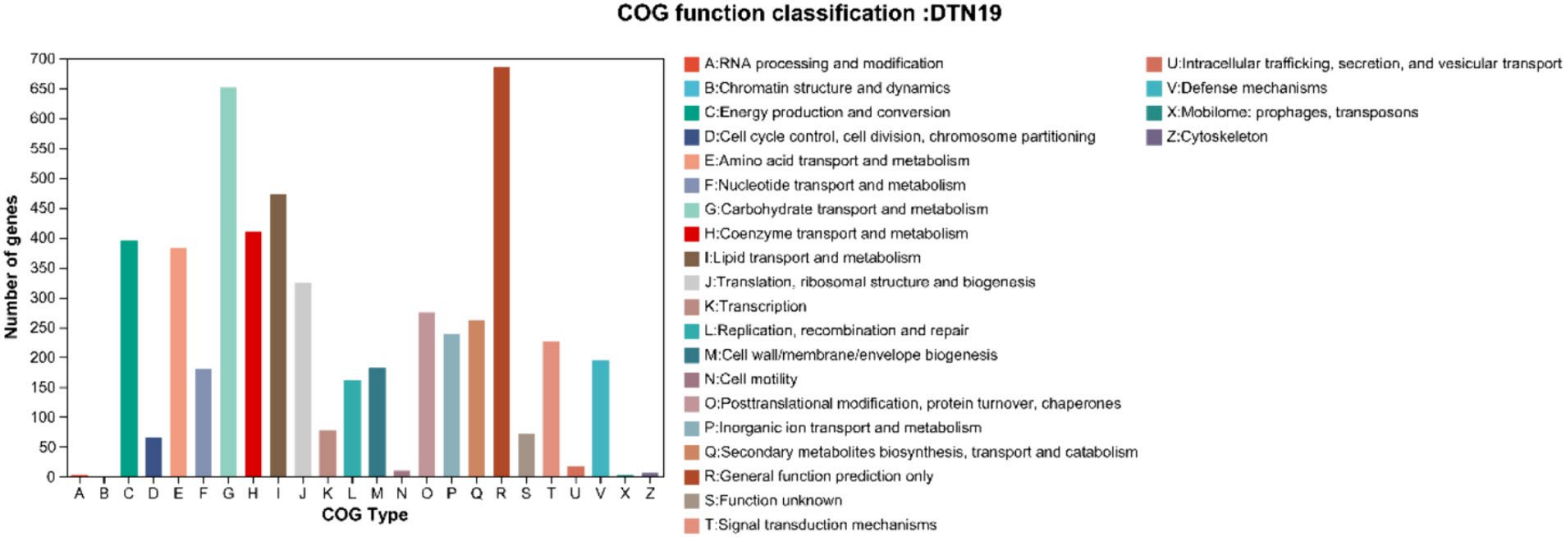
COG function classification of strain DTN19.

Gene annotation and COG prediction for strain DTN19 using the eggNOG database identified a total of 4,453 genes, classified into 25 categories (Figure 8). Excluding the “unknown function” category, the most represented categories are General function prediction (R) with 686 genes; Carbohydrate transport and metabolism (G) with 653 genes; Lipid transport and metabolism (I) with 472 genes; Coenzyme Transport and Metabolism (H) with 472 genes; and Energy production and conversion (c) with 395 genes.

#### 3.6.5 Gene analysis of carbohydrate active enzymes

**Figure 9:**
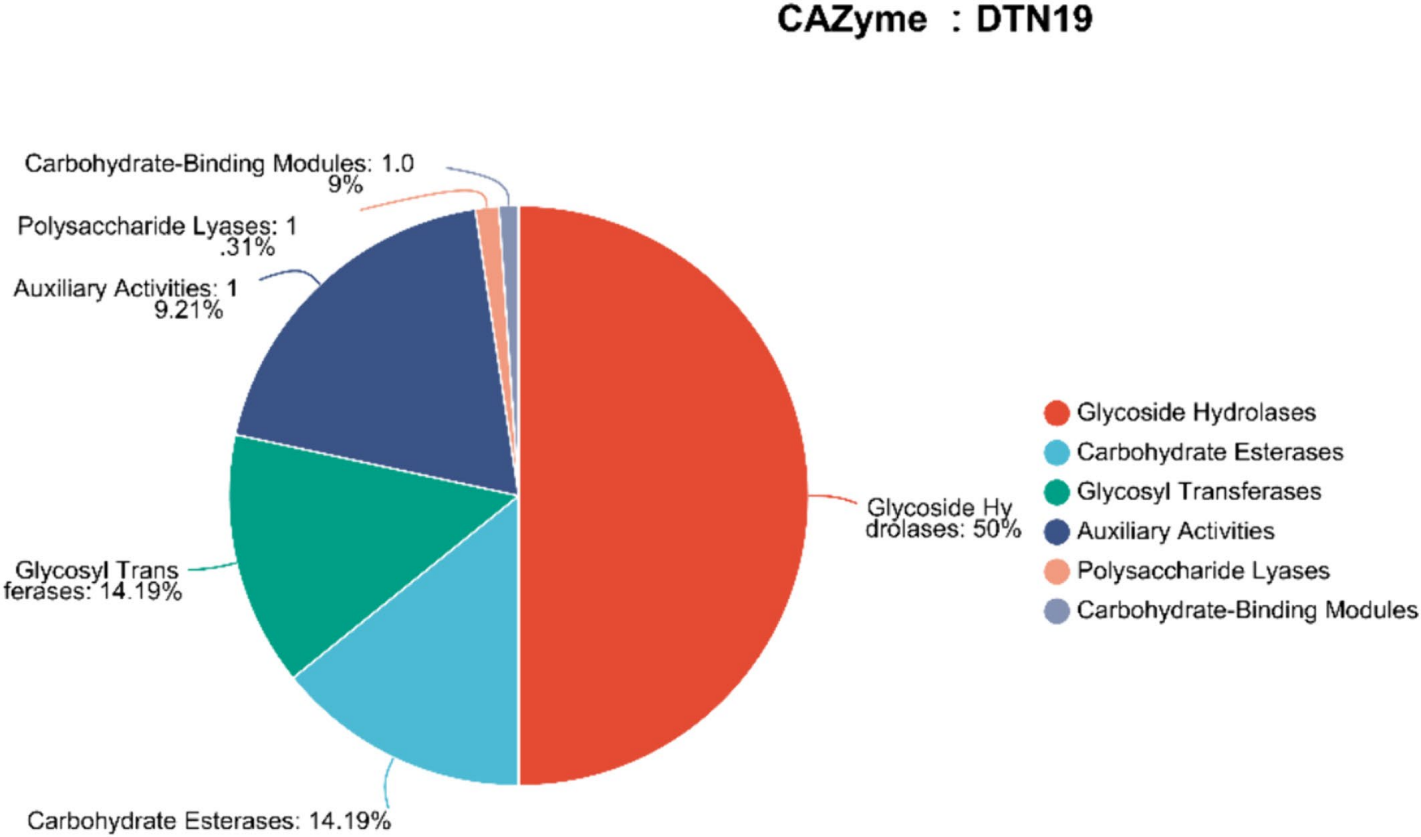
Number of CAZyme genes in DTN19 genome.

Using hmmscan software to predict the CAZy enzyme genes in the genome of strain DTN19, we identified 229 glycoside hydrolases genes, 88 auxiliary activities genes, 65 glycosyl transferases genes, 65 carbohydrate esterases genes, 6 polysaccharide lyases genes, and 5 carbohydrate-binding modules genes (Figure 9).

#### 3.6.6 Prediction of secondary metabolite synthesis gene cluster

Secondary metabolite synthesis gene clusters in strain DTN19 were predicted using the tool available at https://fungismash.secondarymetabolites.org/, identifying a total of 54 clusters (Supplementary Table S4). There are 5 types of these gene clusters, of which T1PKS (type I polyketide synthase) has 17 types. Secondly, there are 11 types of NRPS, 10 types of t1pks-nrps, and 8 types of terpene, which shows that strain DTN19 has the potential ability to synthesize a variety of bioactive substances and has great development value.

## 4 Discussion

Previous studies have reported that non-pathogenic fungi, such as *Trichoderma* and *F. oxysporum* (the main pathogenic fungus) can co-evolve in saffron root soil. In this study, a strain DTN19 with significant antagonistic effects against four saffron pathogens was isolated from healthy saffron rhizosphere soil using a double culture method. We found that *T. brevicompactum* DTN19 not only competes for nutrients and space with pathogenic fungi but also indirectly inhibits their growth through multiple mechanisms. Evaluations of its plant growth-promoting (PGP) performance revealed that DTN19 produces iron carriers and indole-3-acetic acid (IAA). Siderophores, which are organic chelating agents with a low molecular weight (500–1,000 Da), The PGP performance and genome analysis of DTN19 identified genes associated with iron production (*mrp*, *efeU*, gene03368) were found. Although nitrogen is about 78% abundant in the earth’s atmosphere, it cannot be directly used by plants. Nitrogen metabolism-related genes *yafV*, *narK*, *cynT*, *glnA*, *asnB*, and *gdhA* were found in the DTN19 genom. IAA is a plant hormone that regulates plant root growth by stimulating the proliferation and elongation of root cells. The *gatA*, and *amiE* involved in regulating the tryptophan-dependent IAA synthesis pathway were found in the DTN19 genom. Numerous studies highlight the beneficial roles of non-toxic fungal strains, particularly *Trichoderma* species, in plant protection, biostimulation, and biofertilization. *Trichoderma* can parasitize host hyphae, secrete extracellular enzymes to degrade cell walls, or produce secondary metabolites to inhibit or kill pathogenic fungi, thereby preventing and controlling plant diseases (Javaid et al., 2021). It can also form beneficial symbiotic relationships with plants, promoting growth and disease resistance (Adnan et al., 2019; Hewedy et al., 2020; Mulatu et al., 2023). In addition, we also found that its genome contains protease (*pepC*, *VPR*). Genes related to cellulase (gene07578), chitinase, and β -glucosidase (*malZ*, *bglX*, *prtC*, *ygjK*). Huang et al. found that *T. brevicompactum* 31,636 can significantly reduce the abundance of pathogenic fungus and increase the fresh weight, plant height, dry weight, root-shoot ratio, and root length of *Atractylodes macrocephala*, thereby promoting plant growth and yield (Huang et al., 2022). Yao et al. found that strain JSHA-CD-1003 could produce trichodermin, and the minimum inhibitory concentration (MIC) was 25 μg/mL, which effectively inhibited the spore germination and mycelium growth of FocTR4, and the control rate of leaf yellowing of banana *Fusarium* wilt was 47.4%. This is similar to Shi’s discovery (Nosir et al., 2011; Shi et al., 2020; Xiajun et al., 2023).

The main pathogen of saffron corm rot also affects Chinese medicinal materials such as *Atractylodes macrocephala*, *Codonopsis pilosula*, *Panax ginseng*, and *Lycium chinense*, often causing devastating disasters in production (Huang et al., 2021; Uwaremwe et al., 2021; Wang et al., 2022). In recent years, due to factors such as climate, soil, and the pathogenetic fungi’s resistance to fungicides, its harm has been increasing. Therefore, screening more biocontrol fungi to construct a synthetic microbial community or developing new antibiotics from fungi has become an important research direction to control crocus corm disease. Similarly, these two methods are also suitable for the prevention and treatment of other medicinal plant diseases. In this study, the secondary metabolites of strain DTN19 were tested *in vitro*, and it was found that the secondary metabolites had a good antibacterial effect. So far, many studies on the control of corm rot by beneficial fungi and improvement of crocus yield and quality have been reported. *Trichoderma asperellum* inhibits the growth of *F. oxysporum*, significantly reduces the incidence of crocus root rot, and reduces the number of days to germination and flowering of the saffron crop. *Trichoderma harzianum* can inhibit the production of fusaric acid mycotoxin secreted by *F.* oxysporum (Nosir et al., 2011). Two strains of the crocus endophytic fungus *Talaromyces* and *Penicillium* increase the biomass of *C. sativus*; induce the biosynthesis and accumulation of its special metabolites (including crocin I and II), and antagonize the growth of pathogenic fungi to prevent corm rot (Du et al., 2023). Future research will focus on isolating, purifying, and identifying these secondary metabolites to develop new antibiotics for corm rot management. Further screening of biocontrol strains and construction of synthetic microbial communities will enhance biocontrol strategies for saffron corm rot.

## Data Availability

The datasets presented in this study can be found in online repositories. The names of the repository/repositories and accession number(s) can be found in the article/Supplementary material.

## Footnotes

^1^ http://www.ncbi.nlm.nih.gov/

^2^ https://www.ncbi.nlm.nih.gov/BLAST
