# Supplementary material for: Antagonism of rhizosphere Trichoderma brevicompactum DTN19 against the pathogenic fungi causing corm rot in saffron (Crocus sativus L.) in vitro
Source: Front Microbiol. 2024 Sep 4;15:1454670. doi: 10.3389/fmicb.2024.1454670 (PMC11408206; doi:10.3389/fmicb.2024.1454670)
Supplement: Supplementary file 1 [file Table_1.docx]

Supplementary Material

# Supplementary Tables

Supplementary Table S1 Inhibitory effect of strain DN19 on four pathogenic fungi

| **active site** | *F. oxysporum* | *F. solani* | *P. citreosulfuratum* | *P. citrinum* |
| --- | --- | --- | --- | --- |
| Volatile metabolites | 52.17 | 51.13 | 39.22 | 48.47 |
| Non-volatile metabolites | 79.51 | 65.67 | 82.76 | 76.27 |
| fermentation broth extract | 41.33 | 37.31 | 30.16 | 25.75 |

Supplementary Table S2 Results of Biocontrol fungi Producing Degrading Enzymes and Promoting Growth

| **Hydrolytic Enzyme Activities and PGP Attributes** | **Result** |
| --- | --- |
| chitinase | − |
| cellulase | + |
| protease | − |
| glucanase | − |
| IAA production | + |
| phosphate solubilization | − |
| NH_3_ production | − |
| ACC deaminase enzyme | − |
| nitrogen fixation | + |
| potassium dissolution | − |
| siderophore production | + |

Supplementary Table S3 Genome information of Biocontrol fungi

|  | DTN19 |
| --- | --- |
| Gene Total Len (bp) | 22637557 bp |
| GC Content in Gene Region (%) | 51.53% |
| Gene No. | 11437 |
| Gene Average Len | 1979.33 bp |
| rRNAs No. | 64 |
| tRNAs No. | 217 |
| 18S rRNA | 7 |
| 28S rRNA | 7 |
| 5S rRNA | 43 |

Supplementary Table S4 Results of the antimicrobial gene clusters in the genome of strain DTN19

| Region | From | To | Gene amount | Type |
| --- | --- | --- | --- | --- |
| Scaffold5 | 383212 | 404933 | 8 | terpene |
| Scaffold2 | 2695900 | 2741492 | 11 | nrps |
| Scaffold6 | 57631 | 126927 | 9 | nrps |
| Scaffold7 | 1363774 | 1466497 | 13 | t1pks-nrps |
| Scaffold8 | 1674496 | 1695985 | 5 | terpene |
| Scaffold9 | 487044 | 546715 | 12 | nrps |
| Scaffold2 | 578958 | 630825 | 16 | t1pks-nrps |
| Scaffold3 | 2577394 | 2598568 | 8 | terpene |
| Scaffold2 | 1370120 | 1460200 | 14 | t1pks-nrps |
| Scaffold9 | 1581247 | 1628909 | 15 | t1pks |
| Scaffold3 | 5922265 | 5970821 | 15 | t1pks |
| Scaffold2 | 6583825 | 6631855 | 15 | t1pks |
| Scaffold3 | 4604452 | 4652173 | 14 | nrps |
| Scaffold3 | 5313424 | 5361017 | 13 | t1pks |
| Scaffold8 | 118727 | 176711 | 17 | t1pks |
| Scaffold2 | 396059 | 439662 | 16 | other |
| Scaffold5 | 18432 | 64755 | 11 | nrps |
| Scaffold1 | 7536569 | 7589568 | 18 | t1pks-nrps |
| Scaffold6 | 242512 | 285813 | 15 | other |
| Scaffold3 | 4990254 | 5011741 | 7 | terpene |
| Scaffold7 | 517082 | 617296 | 20 | t1pks-nrps |
| Scaffold3 | 5975107 | 6038796 | 17 | nrps |
| Scaffold5 | 576711 | 619936 | 17 | other |
| Scaffold2 | 5616227 | 5637822 | 5 | terpene |
| Scaffold3 | 6240244 | 6297082 | 12 | nrps |
| Scaffold7 | 230914 | 277759 | 18 | t1pks |
| Scaffold4 | 3691220 | 3734720 | 14 | other |
| Scaffold9 | 654527 | 699995 | 13 | t1pks |
| Scaffold7 | 1770460 | 1813549 | 9 | other |
| Scaffold2 | 4612119 | 4661176 | 17 | t1pks |
| Scaffold1 | 155285 | 201930 | 16 | nrps |
| Scaffold2 | 4015319 | 4135390 | 34 | t1pks-nrps |
| Scaffold3 | 2718177 | 2762155 | 17 | other |
| Scaffold1 | 603224 | 654382 | 14 | t1pks |
| Scaffold1 | 1211627 | 1260199 | 15 | t1pks |
| Scaffold1 | 7009895 | 7032039 | 7 | terpene |
| Scaffold1 | 7267832 | 7365103 | 29 | t1pks-nrps |
| Scaffold3 | 1961817 | 2005668 | 12 | other |
| Scaffold3 | 4699063 | 4746227 | 13 | t1pks |
| Scaffold4 | 61614 | 114280 | 11 | t1pks-nrps |
| Scaffold6 | 161492 | 206963 | 15 | nrps |
| Scaffold1 | 6578803 | 6625748 | 13 | t1pks |
| Scaffold1 | 7634627 | 7683329 | 14 | t1pks |
| Scaffold2 | 5969691 | 5991043 | 7 | terpene |
| Scaffold2 | 6275507 | 6323512 | 14 | t1pks |
| Scaffold3 | 4213564 | 4257410 | 14 | other |
| Scaffold7 | 825421 | 846838 | 8 | terpene |
| Scaffold4 | 2259884 | 2306499 | 13 | t1pks |
| Scaffold5 | 270640 | 323196 | 14 | t1pks-nrps |
| Scaffold2 | 4712380 | 4760224 | 14 | t1pks |
| Scaffold5 | 163595 | 262161 | 20 | t1pks-nrps |
| Scaffold5 | 450395 | 505082 | 11 | nrps |
| Scaffold7 | 2217572 | 2266725 | 15 | nrps |
| Scaffold8 | 1899813 | 1945304 | 14 | other |
